# IgG4-Related Perineural Disease

**DOI:** 10.1155/2012/401890

**Published:** 2012-03-18

**Authors:** Dai Inoue, Yoh Zen, Yasuharu Sato, Hitoshi Abo, Hiroshi Demachi, Akio Uchiyama, Toshifumi Gabata, Osamu Matsui

**Affiliations:** ^1^Department of Radiology, Kanazawa University Graduate School of Medical Science, Kanazawa 920-8641, Japan; ^2^Department of Radiology, Toyama Prefectural Central Hospital, Toyama 930-8550, Japan; ^3^Institute of Liver Studies, King's College Hospital, London 5E5 9RS, UK; ^4^Department of Pathology, Okayama University Graduate School of Medicine, Dentistry and Pharmaceutical Sciences, Okayama 700-8558, Japan; ^5^Department of Pathology, Toyama Prefectural Central Hospital, Toyama 930-8550, Japan

## Abstract

*Aims*. To elucidate characteristics of IgG4-related disease involving the peripheral nervous system. *Methods*. Retrospective review of 106 patients with IgG4-related disease identified 21 peripheral nerve lesions in 7 patients. Clinicopathological and radiological features were examined. *Results*. Peripheral nerve lesions were commonly identified in orbital or paravertebral area, involving orbital (*n* = 9), optic (*n* = 4), spinal (*n* = 7), and great auricular nerves (*n* = 1). The predominant radiological feature was a distinct perineural soft tissue mass, ranging 8 to 30 mm in diameter. Histologically, the epineurium was preferentially involved by massive lymphoplasmacytic infiltration rich in IgG4^+^ plasma cells. All lesions were neurologically asymptomatic and steroid-responsive at the first presentation, but one recurrent lesion around the optic nerve caused failing vision. *Conclusion*. IgG4-related disease of the peripheral nervous system is characterized by orbital or paravertebral localization, perineural mass formation, and rare neurologic symptoms. The term “IgG4-related perineural disease” seems appropriate to describe this entity.

## 1. Introduction

IgG4-related disease is a newly designated disease entity, which can be defined as an idiopathic fibroinflammatory condition rich in IgG4^+^ plasma cells. This disease affects a variety of organs including the salivary gland [1], pancreas [2], bile duct [3], lung [4], kidney [5], and aorta/artery [6, 7]. IgG4-related disease shares clinicopathological characteristics irrespective of the affected organs. Clinical features can be summarized as occurring predominantly in adult male patients, elevated serum IgG4 concentrations, responsive to steroid therapy, and synchronous or metachronous association with IgG4-related disease in other organs [8, 9]. IgG4-related disease is histologically characterized by diffuse lymphoplasmacytic infiltration rich in IgG4^+^ plasma cells, storiform fibrosis, obliterative phlebitis, and moderate tissue eosinophilia [1, 3–6, 10]. IgG4-related disease predominantly develops in glandular organs, but nonglandular tissue like retroperitoneum can be affected as well [11, 12].

The diagnosis of IgG4-related disease needs a multidisciplinary approach, in which radiological examination plays an important role. Unique imaging features of IgG4-related disease are of help in recognizing this disease. Radiological examination is regarded as a necessary component for the diagnosis of type 1 autoimmune pancreatitis [13, 14]. Imaging features of renal, pulmonary, or arterial lesions have been also well characterized [15–18]. Recently, a few papers have described the peripheral nerve involvement in IgG4-related disease [19–21]. However, the radiological features of IgG4-related peripheral nerve lesions remain to be clarified.

In this study, we retrospectively examined IgG4-related disease involving the peripheral nervous system. The purpose of this study is to elucidate the clinicopathological and radiological characteristics of IgG4-related peripheral nerve lesions.

## 2. Patients and Methods

### 2.1. Case Selection

This retrospective study was approved by the institutional review board, and the informed consent requirement was waived. We selected consecutive 105 patients (87 men and 18 women; median 68 years, range 38–86 years) that showed radiological features consistent with IgG4-related disease in our hospitals and related institution in the period between September 1998 and May 2011. Another case with IgG4-related peripheral nerve disease (case 6) was obtained from a radiology consultation file. The hospitals involved in this study are university or community-based general hospitals, which have departments of rheumatology, gastroenterology, ophthalmology, otolaryngology, and nephrology so that patients suspected of having IgG4-related disease were referred there. Doctors including radiologists in those hospitals have sufficient knowledge of IgG4-related disease. For most patients, radiological examination first raised a possibility of IgG4-related disease, which was confirmed by serological or pathological examination. Minor exceptions were patients presented with bilateral proptosis with or without submandibular gland enlargement, in which IgG4-related disease was suspected on physical examinations.

We retrospectively reviewed radiology and pathology data of 106 patients with IgG4-related disease collected regarding the presence or absence of macroscopic peripheral nerve abnormalities. A total of 7 patients with peripheral nerve involvement were found and enrolled in this study. All patients were male with a median age of 58 years (range 44–74 years). Clinical features of these patients are summarized in Table 1. Followup data were also examined particularly in terms of the presence or absence of recurrences and neurological symptoms by reviewing inpatient and outpatient records and followup imaging.

### 2.2. Diagnosis of IgG4-Related Disease

The diagnosis of IgG4-related disease was made based on serological, imaging, and histological examinations. Serum IgG4 concentrations were elevated in all patients (median 1280 mg/dL; range 325–3440 mg/dL, normal range <135 mg/dL). Five patients (cases 3–7) had histological examination for specimens taken from the lacrimal gland (surgical biopsy; cases 3, 4, and 6), kidney (needle biopsy; cases 5, 6, and 7), hepatic mass (needle biopsy; case 5), and cervical lymph node (surgical biopsy; case 6). Cases 1 and 7 had surgical biopsies from peripheral nerve lesions. Case 2, who did not have histological examination, was diagnosed as IgG4-related ophthalmic disease based on the clinical presentation with left proptosis, imaging features, and a high serum IgG4 concentration (372 mg/dL).

All resected or biopsied specimens were reviewed by a pathologist and confirmed features consistent with IgG4-related disease including diffuse lymphoplasmacytic infiltration, storiform fibrosis, obliterative phlebitis, occasional eosinophils, numerous IgG4^+^ plasma cell infiltrates, and high IgG4^+^/IgG^+^ plasma cell ratios (>40%) [1, 3, 5, 6].

### 2.3. Radiological Examinations

All images were reviewed by two radiologists, and decisions were reached by consensus. Because of the retrospective nature of this study, the imaging examinations performed were not consistent. Imaging examinations evaluable for neural lesions performed were CT in six patients (cases 1–6), MRI in five (cases 1–4, 6), and 18F-fluorodeoxyglucose positron emission tomography (FDG-PET) in one (case 5). Case 7 did not undergo radiological examination for the neural lesion. Images were reviewed in terms of the location, shape, and size of each lesion, configuration of the border to surrounding adipose tissue (well circumscribed or infiltrative), and the presence or absence of radiologically detectable nerve fibers within the lesion. The size of lesions was defined as the maximum diameter measured in the vertical imaging plane to the peripheral lesions.

## 3. Results

### 3.1. Clinical Characteristics

#### 3.1.1. Location

A total of 21 peripheral nerve lesions were identified in 7 patients. Six of them were found in a consecutive cohort of 105 patients with IgG4-related disease in radiology database, suggesting the prevalence of the peripheral nerve involvement in IgG4-related disease to be 5.8% with a 95% confidence interval of 2.1% to 12.0%. For the remaining one, only clinicopathological and radiology data were provided for a second opinion from a referring hospital. Locations and radiological features are summarized in Table 3. Of 7 patients, 5 (71%) had two or more lesions simultaneously. Twenty lesions (95%) were located in orbital or paravertebral area, involving infraorbital (*n* = 5), supraorbital (*n* = 4), optic (*n* = 4), lumbar spinal (*n* = 3), sacral spinal (*n* = 3), and cervical spinal nerves (*n* = 1). The remaining lesion was involvement of great auricular nerve in a cervical mass in case 7. Peripheral nerve lesions in ophthalmic area were always associated with IgG4-related dacryoadenitis or orbital disease, whereas spinal nerve lesions were isolated without IgG4-related disease in adjacent tissue. Among the consecutive cohort of 105 patients, 18 patients (17%) had ophthalmic lesions and 6 of them (6/18) appeared to have peripheral nervous involvement, suggesting the prevalence of 33.3% with a 95% confidence interval of 13.3% to 59.0%.

#### 3.1.2. Other Organs Involvement

All patients were found to have IgG4-related lesions in other organs, all but one of which were identified at the same time as the neural lesions (Table 2). Enlargement of bilateral lacrimal glands, which had been present for 10 years before the episode of the peripheral nerve lesion, in case 7 was retrospectively diagnosed as IgG4-related dacryoadenitis.

#### 3.1.3. Symptoms

All patients presented symptomatically (Table 1). Symptoms were related to other organ lesions or mass effects of peripheral nerve lesions. Real neurological signs such as paralysis were not evident in any cases at the first presentation. Interestingly, cases 1, 2, and 4 had double vision due to abnormal ocular movement. This attributed to mass effects of IgG4-related ophthalmic disease including dacryoadenitis, myossitis, and perineural lesions, because peripheral nerve lesions in these patients involved supraorbital, infraorbital, and optic nerves, all of which are sensory nerves unlikely to cause abnormal ocular movement. The cause of epigastralgia in case 5 was not identified by serological and endoscopic examinations. This symptom spontaneously resolved.

#### 3.1.4. Treatment and Recurrence

All patients had followup images of at least either CT or MRI for peripheral nerve lesions. Both chest and abdominal CTs, which were also available for all patients, were reviewed with regard to recurrent lesions at other sites. Steroid therapy at an initial dose of 20 to 40 mg/day was effective for all patients, making peripheral nerve lesions decrease in size in conjunction with decreased size of other organ lesions. Recurrent perineural lesions were confirmed during steroid taper in two patients (cases 2 and 4). In case 2, the recurrent lesion, which compressed the left optic nerve in the optic bony canal, caused failing vision and papilledema. The recurrent lesion promptly responded to an increased dose of steroid. Visual acuity and papilledema were fully recovered. Although the perineural mass focally remained in the most recent followup MRI, he is currently free of neurological symptoms. Recurrent lesions involving right optic and infraorbital nerves in case 4 also decreased in size with an increased dose of steroid.

### 3.2. Imaging Characteristics

**Figure 1:**
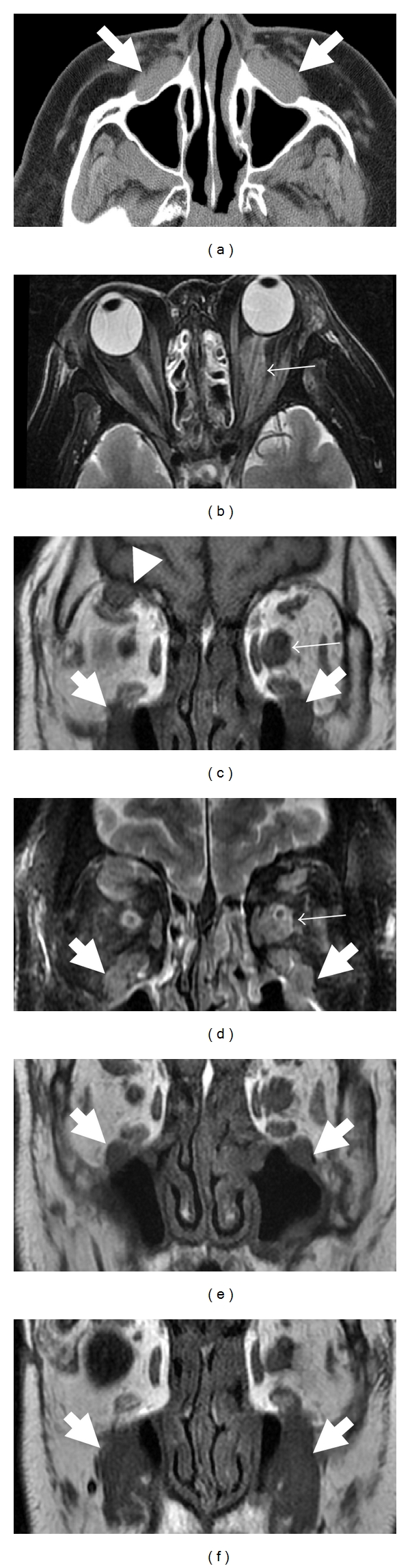
CT (a) and MRI ((b and d): T2-weighted images; (c, e, and f): T1-weighted images) of orbital space in 55-year-old man (case 1). CT image reveals soft tissue around the bilateral infra orbital nerve (a; arrows). MRI shows soft tissue around the left orbital nerve (b–d; small arrows), bilateral infraorbital nerve (c–f; arrows) and right supraorbital nerve (c and d; arrowheads). Left orbital nerve can be detected in the lesion (b and d).

**Figure 2:**
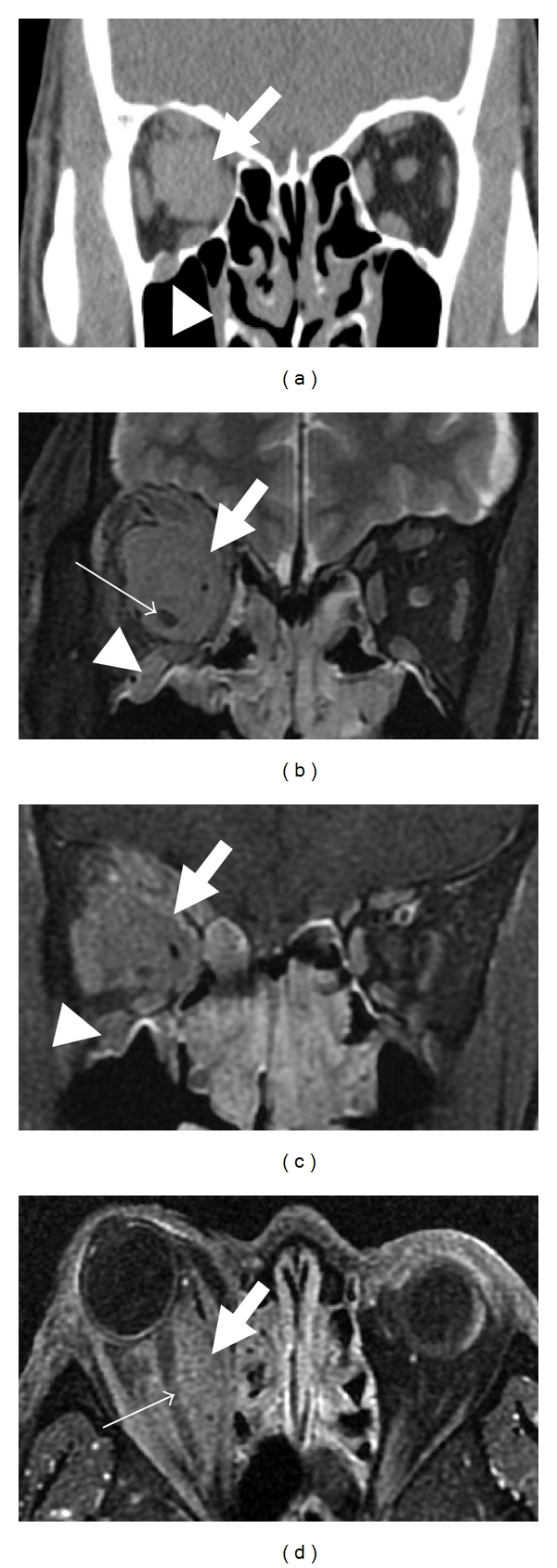
CT (a) and MRI (b: T2-weighted image; (c and d): contrast-enhanced T1-weighted images) of orbital space in 44-year-old man (case 4). Soft tissue mass is detected in the right orbital space (a; arrow). This lesion extends along the right optic nerve in MRI (b–d; arrows). The right optic nerve penetrating the lesion is detectable in MRI (b and d; small arrows). Soft tissue mass along the right infraorbital nerve is also noted (a–c; arrowheads).

**Figure 3:**
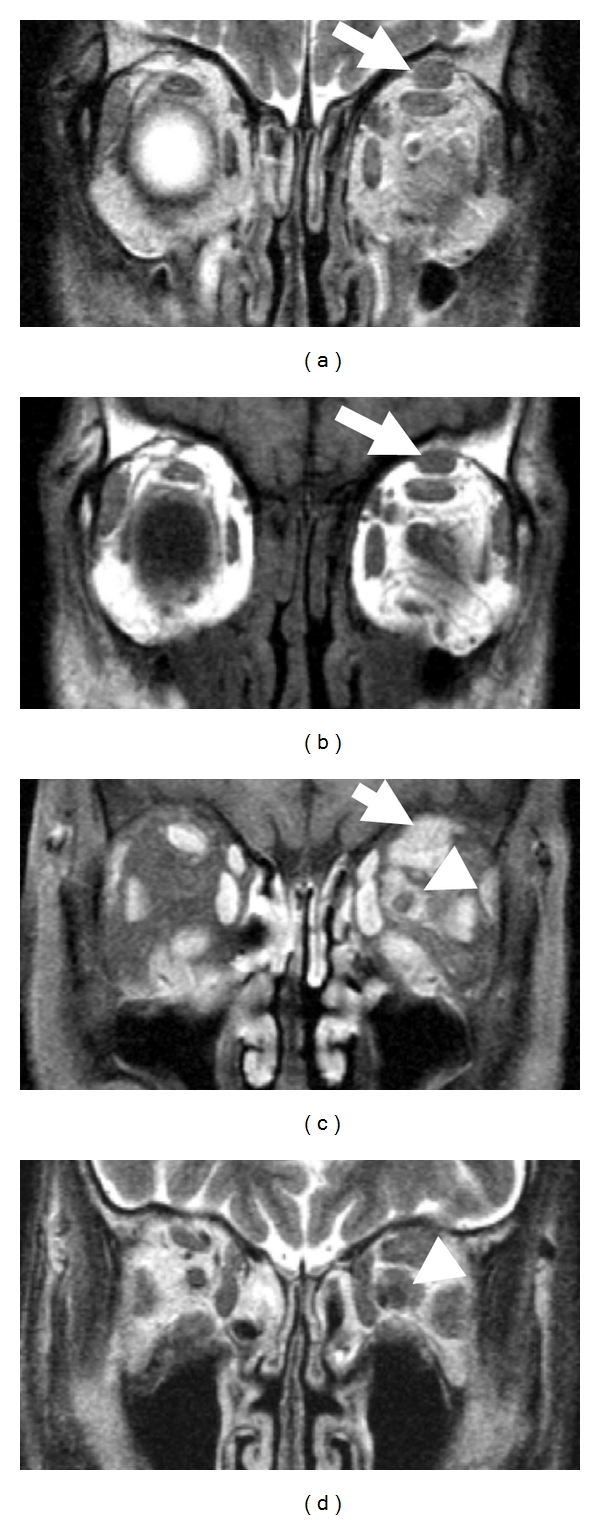
MRI ((a and d): T2-weighted images; b: T1-weighted image; c: contrast-enhanced T1-weighted image) of orbital space in 61-year-old man (case 3). MRI shows the soft tissue around the left supraorbital nerve (a–c; arrows) and optic nerve (c and d; arrowheads). The left optic nerve can be detected in the lesion.

**Figure 4:**
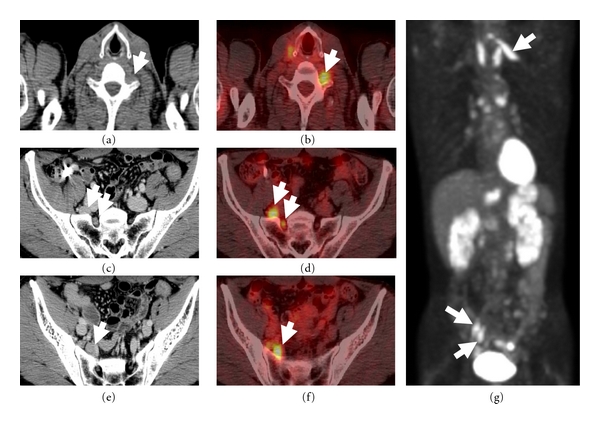
CT (a, c, and e) and FDG-PET (b, d, and f: fusion images; g: coronal MIP image) of whole body in 58-year-old man (case 5). CT shows soft tissue density around the left C6 (a; arrow), right L5 (c; arrows), and S1 nerves (e; arrow). High FDG uptakes are identified as perineural masses (b, d, f, and g; arrows).

Radiological examination was performed for peripheral nerve lesions in 6 patients (cases 1–6). All lesions were radiologically characterized by distinct masses along the affected nerve fascicles (Figures 1–4). The size of the lesions ranged from 8 to 30 mm (median 13 mm). In case 1, bilateral orbital masses extended to the subcutis of the cheek along infraorbital nerves and their branches (Figures 1(a), 1(c)–1(f)). Spinal nerve lesions in cases 5 and 6 involved nerve fascicles mainly within and distal to intervertebral foramen, and portions proximal to the foramen, which anatomically correspond to nerve roots, were affected only in cervical nerve lesion of case 5. All lesions were well circumscribed with a round or lobular shape. The latter was common in optic nerve lesions (Table 3). On CT images, all lesions showed isodensity compared with those of skeletal muscles. Peripheral nerve lesions showed isointensity in T1-weighted MRI images and iso- to slightly high-intensity in T2-weighted images compared with those of skeletal muscles (Figures 1–4). All lesions were homogeneously enhanced (Figures 2(c), 2(d), and 3(c)). Calcification or necrosis was not a feature in any lesion.

Although nerve fibers involved in masses were not identifiable in CT images (Figure 2(a)), MRI demonstrated optic nerves penetrating the lesions in cases 1, 2, 3, and 4 (Figures 1(b), 1(d), 2(b), 2(d), and 3(c)). Intralesional nerves did not show any abnormalities regarding the signal intensity or pattern of contrast enhancement. FDG-PET, which was performed in only case 5, showed high uptakes in perineural lesions with SUV max 5.6 in C6, 5.2 in right L5, and 4.6 in right S1 (Figures 4(b), 4(d), 4(f), and 4(g)).

### 3.3. Histopathological Features of the Perineural Lesions

Histopathological examinations in perineural lesions were performed in cases 1 and 7. Case 1 had a surgical biopsy from the peripheral nerve lesion involving the infraorbital nerve. The pathology specimen consisting of perineural soft tissue showed severe lymphoplasmacytic infiltration, irregular fibrosis, and occasional eosinophils. Obliterative phlebitis was not identified. Immunostaining revealed a large number of IgG4^+^ plasma cells (107 cells/hpf (0.237 square mm)) and an IgG4/IgG ratio was 61.8%. The relationship between nerve fascicles and inflammation could not be assessed because no nerve fibers were sampled.

**Figure 5:**
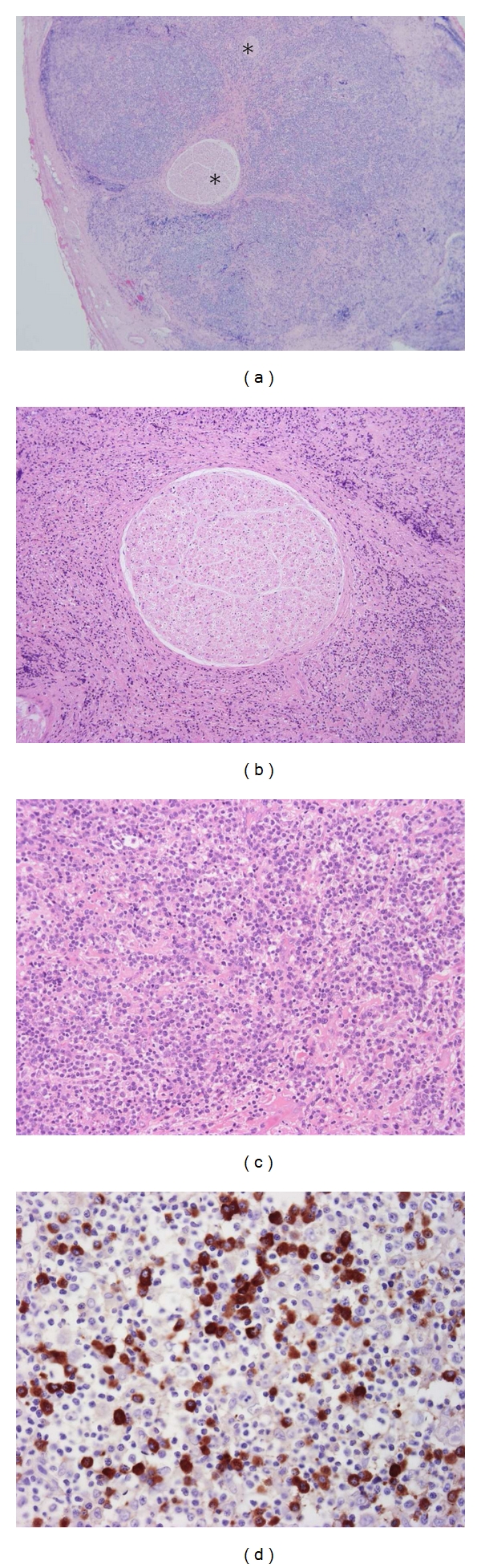
Histological features of resected IgG4-related perineural disease in 61-year-old man (case 7). (a) The epineurium is involved in a massive inflammatory process, where nerve fascicles (*) are embedded. (b) The endoneurium is unremarkable without inflammatory cell infiltration. (c) Inflammatory cells consist predominantly of lymphocytes and plasma cells. (d) Immunostaining for IgG4 reveals a large number of IgG4+ plasma cells.

Case 7, who did not undergo radiological examination of the peripheral nerve lesion, had IgG4-related disease in bilateral lacrimal glands, lung (interstitial pneumonia), and kidney. He noticed a palpable mass in the left neck, which was surgically resected on suspicion of an enlarged lymph node. Macroscopically, the resected lesion was a well-circumscribed spherical mass measuring 15 mm in the maximum diameter. Histologically, the specimen was not a lymph node but an inflammatory nodule centered on large nerve fascicles (Figures 5(a) and 5(b)), which were considered to be the great auricular nerve. The epineurium was extensively enlarged with massive lymphoplasmacytic infiltration rich in IgG4^+^ plasma cells (IgG4^+^ plasma cells 215 cells/hpf; IgG4/IgG 58.9%) (Figures 5(c) and 5(d)). Fibrosis or eosinophilic infiltration was not conspicuous. Penetrating nerve fibers, which were separated from the inflammatory process by the perineurium, were histologically unremarkable with scarce intraneural inflammatory cell infiltration (Figure 5(b)). He eventually noticed sensory dysfunction in the left retroauricular region after the surgical biopsy probably because the nerve was surgically transected.

Compared to radiological features and histological findings, perineural masses corresponded to the expanded perineurium affected by the IgG4-related inflammatory process. Homogeneous enhancement on imagings was supposed to represent massive inflammatory cell infiltration in the perineurium.

## 4. Discussion

The obtained results can be summarized as follows: (1) IgG4-related disease can rarely involve peripheral nerves particularly in ocular or paravertebral area, the former usually associated with ophthalmic lesions. (2) IgG4-related neural lesions are radiologically characterized by nerve-centered distinct soft tissue masses. (3) Histological features are massive lymphoplasmacytic infiltrates rich in IgG4^+^ plasma cells predominantly affecting the epineurium, suggesting “IgG4-related perineural disease” to be an appropriate term to describe these lesions. (4) Neurological symptoms are only rarely noted in this retrospective study.

This study demonstrated that the peripheral nervous system is one of the organs that can be involved in IgG4-related disease. It is unclear how IgG4-related perineural disease was interpreted before the entity of IgG4-related disease was recognized. Orbital lesions that involve both peripheral nerves and adjacent ophthalmic tissue might be referred to as orbital inflammatory pseudotumors [22]. Perineuritis is another possibility particularly for an isolated perineural disease as seen in cases 2 and 7.

The predominant radiological feature of IgG4-related perineural disease was a well-circumscribed mass, seen as soft tissue intensity on MRI. Interestingly, MRI demonstrated unremarkable optic nerves penetrating the perineural masses, suggesting little, if any, damage to the nerve fascicles themselves. This is in keeping with the fact that nerve fibers entrapped in the lesion were histologically unremarkable and neurological symptoms were rare. However, smaller nerves, such as the infraorbital nerve, were difficult to visualize on MRI. It is not yet conclusive to what extent FDG-PET is useful in detecting IgG4-related perineural disease like as reported in other organs [23], but this functional imaging helped us to recognize spinal nerve lesions, which were overlooked by other modalities, in case 5.

IgG4-related perineural disease needs to be differentiated from a variety of other diseases including malignant lymphoma, neurolymphomatosis [24], neurogenic tumors, and non IgG4-related inflammatory diseases such as sarcoidosis [25] or idiopathic inflammatory pseudotumor. The presence or absence of IgG4-related disease in other organs seems most helpful for this differential diagnosis, given that all cases in this study had other organ manifestations. Serum IgG4 concentrations are also useful for the diagnosis, as is true with IgG4-related disease in other organs. However, histological examination is still necessary for patients with unusual clinical or radiological features. It is still an open question whether IgG4-related perineural disease is always associated with other organ lesions or can develop as a solitary IgG4-related lesion.

It is interesting that 13 of 21 (62%) perineural lesions were seen in the orbital area, where branches of the trigeminal nerve were commonly involved (43%). Affected nerves described in previous papers were also trigeminal or optic nerve branches [19–21]. One possible explanation of this site predilection is that IgG4-related ophthalmic disease more commonly shows perineural extension than other organ manifestations. In fact, all ophthalmic perineural lesions were associated with IgG4-related orbital disease. Ophthalmic perineural lesions were identified in 33% (6/18) of patients with IgG4-related orbital disease in our cohort. Another interesting point is that all perineural lesions identified were rather proximal and localize to where nerves encounter foramen. Molecules that can be targeted by IgG4-related disease may be more abundantly present in proximal parts of peripheral nerves. Another possible explanation is that distal perineural masses may be difficult to recognize as being along small nerves leading to false recognition as other anatomical structures like enlarged lymph nodes on images. In fact, microscopic perineural inflammation is commonly seen in surgically resected specimens of IgG4-related disease.

This study had a few limitations. Because of the retrospective nature of this study, imaging examinations underwent for each cases were not consistent. Neurological symptoms might not be extensively examined because a neurological involvement was not suspected at diagnosis. A prospective study seems necessary to conclude how often and to what extent IgG4-related perineural disease can cause neurological symptoms. Someone might also argue that IgG4-related inflammation was confirmed in only 2 cases. However, perineural lesions in the other patients most likely attributed to IgG4-related disease given the fact that they were present at diagnosis and responded to steroid therapy and the radiological features would not fit with other known neuropathies.

In conclusion, IgG4-related disease of the peripheral nervous system, which can be called IgG4-related perineural disease, is characterized by orbital and paravertebral localization, perineural mass formation detectable as soft tissue intensity in MRI images, and rare neurological symptoms.

## Figures and Tables

**Table 1:** Clinical characteristics, laboratory data, and initial dose of steroid.

Case	Age	Gender	Symptom	IgG (mg/dL)*	IgG4 (mg/dL)^†^	ANA (titer)	IgE (IU/mL)^‡^	Initial dose of steroid
1	55	Male	Double vision, subcutaneous nodule in cheek	4845	2050	×40	988	30 mg/day
2	53	Male	Left proptosis, double vision	NA	372	×40	NA	30 mg/day
3	61	Male	Lacrimal gland enlargement	1450	463	<40	4608	20 mg/day
4	44	Male	Right proptosis, double vision, lacrimal gland enlargement	1146	325	<40	151	30 mg/day
5	58	Male	Epigastralgia	2850	1280	NA	NA	30 mg/day
6	74	Male	Lacrimal gland enlargement, cervical lymph node enlargement	6024	2550	NA	NA	30 mg/day
7	61	Male	Palpable left cervical mass, lacrimal gland enlargement,	7364	3440	×2560	1146	40 mg/day

NA: not analyzed; *normal range < 1600 mg/dL; ^†^normal range < 135 mg/dL; ^‡^normal range < 170 IU/mL.

**Table 2:** IgG4-related disease identified in other organs.

Case	Other organ manifestations
1	Enlargement of the left extraocular muscle
2	Enlargement of the left extraocular muscle
3	Dacryoadenitis, sialadenitis
4	Dacryoadenitis
5	Hepatic inflammatory pseudotumor, tubulointerstitial nephritis
6	Dacryoadenitis, mediastinal lymphadenopathy, tubulointerstitial nephritis, enlargement of the bilateral extra-ocular muscle
7	Dacryoadenitis,*lung lesions, tubulointerstitial nephritis

*A single lesion identified before the episode of perineural disease.

**Table 3:** Characteristics of perineural lesions.

Case	Affected nerve	Performed imaging examinations	Size	Shape	Involved nerve
1	Right supraorbital nerve	CT (P), MRI (P)	15 mm	Round	Not visible
	Right infraorbital nerve	CT (P), MRI (P)	25 mm	Lobular	Not visible
	Left infraorbital nerve	CT (P), MRI (P)	26 mm	Lobular	Not visible
	Left optic nerve	CT (P), MRI (P)	12 mm	Lobular	Identifiable (MRI)
2	Left optic nerve	CT (P), MRI (CE)	14 mm	Lobular	Identifiable (MRI)
3	Left supraorbital nerve	CT (P), MRI (P)	10 mm	Round	Not visible
	Left optic nerve	CT (P), MRI (P)	10 mm	Lobular	Identifiable (MRI)
4	Right infraorbital nerve	CT (P), MRI (CE)	8 mm	Round	Not visible
	Right optic nerve	CT (P), MRI (CE)	30 mm	Lobular	Identifiable (MRI)
5	Left C6 nerve	CT (P), FDG-PET	9 mm	Round	Not visible
	Right L5 nerve	CT (CE), FDG-PET	13 mm	Round	Not visible
	Right S1 nerve	CT (CE), FDG-PET	13 mm	Round	Not visible
6	Right supraorbital nerve	CT (P), MRI (CE)	8 mm	Round	Not visible
	Right infraorbital nerve	CT (P), MRI (CE)	12 mm	Round	Not visible
	Left supraorbital nerve	CT (P), MRI (CE)	13 mm	Round	Not visible
	Left infraorbital nerve	CT (P), MRI (CE)	12 mm	Round	Not visible
	Right L5 nerve	CT (CE)	14 mm	Round	Not visible
	Left L5 nerve	CT (CE)	19 mm	Round	Not visible
	Right S1 nerve	CT (CE)	9 mm	Round	Not visible
	Left S1 nerve	CT (CE)	13 mm	Round	Not visible
7	Left greater auricular nerve	None	15 mm*	Round*	Not analyzed

P: plain; CE: contrast-enhanced; *macroscopically examined on the resected specimen.

## Abbreviations

CT: Computed tomography
MRI: Magnetic resonance imaging
FDG-PET: 18F-fluorodeoxyglucose positron emission tomography
H&E: Hematoxylin and eosin.
